# A Curious Case of Leg Swelling With Eosinophilic Fasciitis, IgA-κ Monoclonal Gammopathy, Proteinuria, and Bilateral Carpal Tunnel Neuropathy: Diagnostic Convergence of Sclerosing Dermato-Fasciitis and Monoclonal Gammopathy

**DOI:** 10.7759/cureus.111358

**Published:** 2026-06-23

**Authors:** Sachin Dave, Vidit Jogani, Raj Palraj, Ala S Dababneh, Mariya Pogorelova, Ibrahim Abubeker

**Affiliations:** 1 General Internal Medicine, Mayo Clinic, Rochester, USA; 2 Medicine, Mayo Clinic, Rochester, USA; 3 General Internal Medicine, Infectious Diseases, Mayo Clinic, Rochester, USA

**Keywords:** atypical leg swelling, eosiniphilia, eosinophilic fasciitis, groove sign, mgus, monoclonal gammopathy, monoclonal gammopathy of undetermined significance (mgus), peau d'orange, protienuria

## Abstract

Eosinophilic fasciitis (EF) is a rare sclerosing disorder characterized by limb edema evolving to woody induration with peau d'orange texture and the groove sign. Diagnosis typically requires a full-thickness fascial biopsy, while magnetic resonance imaging (MRI) can noninvasively demonstrate fascial thickening and guide biopsy. EF has recognized associations with hematologic conditions, including monoclonal gammopathy. A previously healthy 64-year-old woman developed progressive lower extremity hyperpigmentation, edema, and painful induration after travel. Over months, she experienced forearm induration and neuropathic hand pain; examination revealed bilateral lower leg peau d'orange and a right forearm groove sign. Laboratory evaluation showed episodic peripheral eosinophilia and an IgA-κ monoclonal gammopathy with nephrotic-range proteinuria. Electromyography confirmed bilateral median neuropathy at the wrists. Empiric prednisone produced marked symptomatic improvement. Bone marrow evaluation was consistent with monoclonal gammopathy of undetermined significance (MGUS). Kidney biopsy showed no amyloid, monoclonal immunoglobulin deposition disease (MIDD), proliferative glomerulonephritis with monoclonal deposits (PGNMID), light-chain cast nephropathy, or light-chain tubulopathy - making monoclonal gammopathy of renal significance (MGRS) unlikely. The hallmark cutaneous signs of EF (groove sign, peau d'orange) and limb-predominant involvement favored EF over mimics such as scleredema, morphea profunda, and systemic sclerosis. MRI is useful to confirm fascial involvement and guide biopsy when patients hesitate about incisional sampling. EF is classically steroid-responsive; methotrexate or mycophenolate are common steroid-sparing agents, with biologics or intravenous immunoglobulin (IVIG) reserved for refractory disease. The patient also had MGUS and carpal tunnel syndrome - both conditions that may raise concern for amyloidosis; however, amyloid was excluded histopathologically. This case highlights EF associated with IgA-κ MGUS and significant proteinuria, in which a kidney biopsy excluded MGRS lesions, illustrating the importance of multidisciplinary evaluation and targeted tissue diagnosis. Prompt recognition of EF's bedside signs can expedite treatment and functional recovery while more invasive diagnostics are considered.

## Introduction

Eosinophilic fasciitis (EF; Shulman syndrome) is a rare fibrosing disorder marked by an edematous inflammatory phase, followed by symmetric induration of the limbs, often sparing the hands and face. Typical bedside signs include peau d’orange and the groove sign, a linear depression along superficial veins accentuated with limb elevation. Peripheral eosinophilia, hypergammaglobulinemia, and elevated ESR are frequent [1,2]. MRI can show fascial thickening and enhancement and is helpful for diagnosis, monitoring, and biopsy site selection, although a full‑thickness wedge biopsy, including fascia, remains the gold standard [3]. EF has been linked to hematologic abnormalities, including monoclonal gammopathy of unknown significance (MGUS) and, rarely, overt malignancy. Recent studies suggest monoclonal gammopathy may be present in a notable subset of EF patients [4]. We report a woman with EF‑consistent findings, IgA‑κ MGUS, heavy proteinuria without kidney monoclonal gammopathy of renal significance (MGRS), and bilateral median neuropathy, underscoring diagnostic intersections between sclerosing dermato‑fasciitis and monoclonal gammopathy [5]. MGRS is defined as a clonal B-cell or plasma-cell disorder that does not meet criteria for overt malignancy but produces a monoclonal immunoglobulin causing kidney injury. MGUS prevalence in EF remains uncertain. Few case reports have highlighted its association. IgA-k MGUS isotype's novel association with EF is highlighted by this case report.

## Case presentation

A 64‑year‑old woman of Serbian ancestry with no significant prior medical history presented with progressive swelling and pain in both legs and hands, and leg hyperpigmentation beginning in October 2023 while traveling internationally. Six months later, the skin started becoming thicker. Symptoms evolved over the next 12 months to severe limb discomfort and fatigue, limiting activities of daily living. She also developed episodic paresthesia in both hands. She presented to our clinic in February 2025 for further evaluation, resulting in outlined testing and diagnosis. On review, she had loud snoring and daytime fatigue, raising concern for obstructive sleep apnea.

Examination

**Figure 1:**
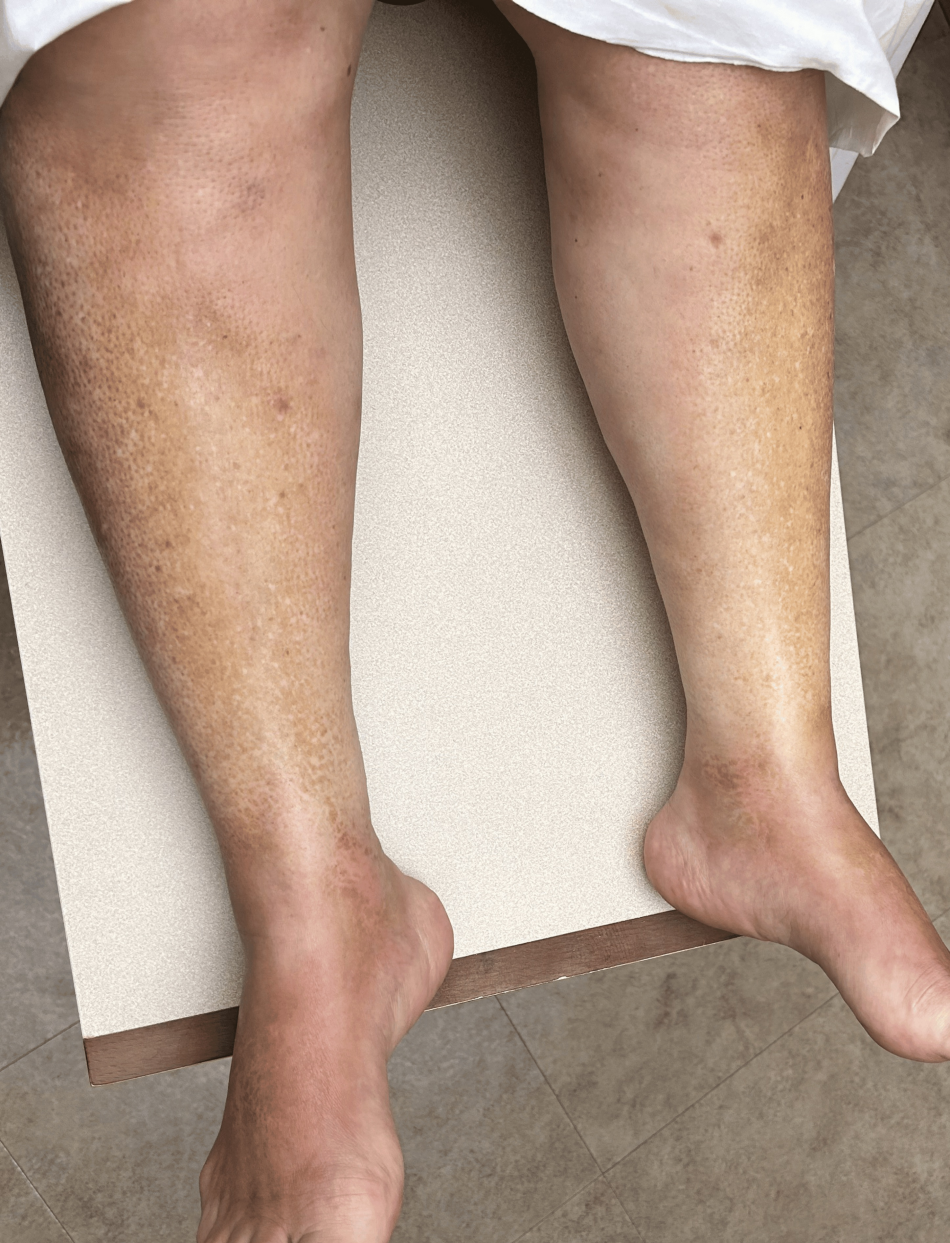
Leg swelling and skin peau d'orange

Her vitals were normal, her head/neck showed a high‑arched palate and crowded oropharynx, and her lungs were clear; a soft systolic ejection murmur was noted. Her abdomen was soft; mild abdominal wall edema was suspected. Both lower legs had woody induration with peau d'orange, hyperpigmentation, and ankle warmth/erythema (Figure 1). There was 1+ pitting edema and firm subcutaneous thickening. Her right forearm had a positive groove sign, and her hands showed soft‑tissue swelling without definite sclerodactyly. There was clear induration of bilateral legs and the right more than the left forearm, equivocal trunk induration, and absence of hand/finger thickening. The groove sign was present.

Laboratory and imaging

CBC revealed eosinophilia; inflammatory markers (ESR and CRP) were elevated. IgG was elevated. Serum protein studies showed IgA‑κ monoclonal gammopathy with elevated free κ light chain and urine protein initially ~3-5 g/day. Right wrist radiographs were unremarkable, serum albumin was 2.8 g/dL, and the estimated GFR was 55 mL/min/1.73 m^2^. Moderately positive anti-nuclear antibody (ANA) in the absence of other positive serologic auto-immune or connective tissue cascade markers was a nonspecific abnormality (Table 1).

**Table 1:** Laboratory and diagnostic findings

Parameter	Patient Value	Reference Range	Units
Absolute Eosinophil Count	0.53	0.0-0.5	× 10⁹ cells/L
ESR	112	<20	mm/hour
CRP	37.1	<5	mg/L
ANA (HEp‑2)	Positive (1:160)	Negative	-
Antinuclear Antibody, Serum	0.3	<1	units
Anti-dsDNA Ab, IgG, Serum	<10	0-99	IU/mL
C4 Complement	23	14-40	mg/dL
C3 Complement	102	75-175	mg/dL
Myeloperoxidase Ab (MPO), Serum	<0.2	<0.4	units
Proteinase 3 Ab (PR3), Serum	<0.2	<0.4	units
Anti-Scl-70	Negative	Negative	-
Anti-CCP	Negative	Negative	-
Rheumatoid Factor	Negative	Negative	-
IgA	512	70-400	mg/dL
IgG	1870	700-1600	mg/dL
IgM	183	40-230	mg/dL
Serum Creatinine	1.12	0.6-1.3	mg/dL
Serum Albumin	2.8	3.4-5.4	g/dL
24-Hour Urine Protein	2976	<150	mg/day
eGFR	55	>90	mL/min/1.73 m²
M-protein (FLAG)	Positive	Absent	-
Bone Marrow Biopsy	MGUS	Normal marrow	-
Amyloidosis	No evidence	Absent	-
Kidney Biopsy	Membranous nephropathy with focal crescentic injury	Normal histology	-

EMG confirmed bilateral median neuropathy at the wrist (carpal tunnel). Given carpal tunnel and monoclonal gammopathy, systemic amyloidosis was considered. Bone marrow findings supported MGUS; kidney biopsy demonstrated the absence of all of the following: amyloid, monoclonal immunoglobulin deposition, proliferative GN with monoclonal deposits, and light‑chain cast nephropathy or tubulopathy. Phospholipase A2 receptor (PLA2R) was equivocal.

MRI of the lower limbs was done to assess fascial involvement and to guide any future biopsy.

Working diagnoses and consultations: The leading consideration was EF, with differential diagnoses including scleroderma (noting the patient's IgA‑κ MGUS), morphea profunda, stiff skin syndrome, and systemic sclerosis. Rheumatology, Dermatology, Nephrology, Hematology, and Infectious Diseases were involved. Patient deferred full-thickness fascial biopsy.

Differential diagnosis

Eosinophilic fasciitis (EF): Supported by limb‑predominant induration, groove sign, peau d'orange, steroid responsiveness, and intermittent peripheral eosinophilia; the hands and face were relatively spared. MRI is expected to show fascial thickening; full‑thickness fascial biopsy remains a diagnostic gold standard (patient hesitant) [1-3].

Scleroderma (type 2, paraproteinemia‑associated): Considered due to IgA‑κ MGUS; however, scleroderma classically involves the upper back/neck and often spares the distal extremities, whereas this patient had distal leg and forearm involvement with groove sign - findings more typical of EF [6].

Morphea profunda: Can overlap clinically and histologically with EF; peripheral eosinophilia and fascial eosinophils favor EF over morphea profunda [2,7].

Systemic sclerosis: Less likely given the absence of Raynaud phenomenon, nailfold capillary changes, internal organ involvement, and sclerodactyly [7].

Amyloidosis: Considered for (a) MGUS with proteinuria and (b) bilateral carpal tunnel as red flags; excluded by kidney and bone marrow biopsy and fat pad aspiration. Carpal tunnel can be an early signal for transthyretin amyloidosis, necessitating vigilance; in this case, tissue studies were negative [8].

Investigations 

**Figure 2:**
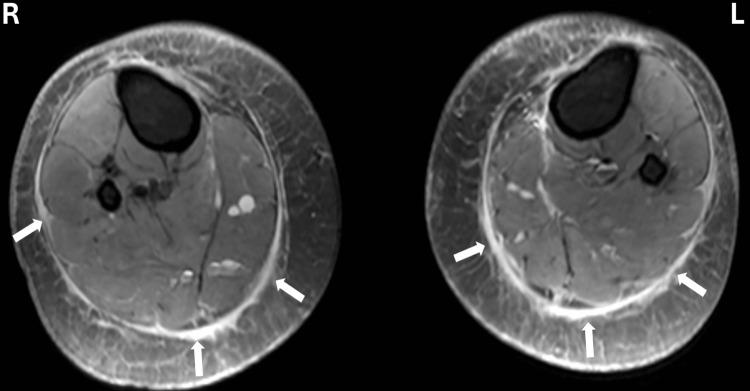
Axial T2 MRI of the lower limbs showing fascial thickening (arrows)

Serology/hematology includes IgA‑κ M protein, elevated free κ light chain, eosinophilia, and normocytic anemia (Table 1). In urine studies, proteinuria was about 3 g in 24 hours, as outlined in Table 1. MRI of the legs showed fascial involvement, as outlined in the diagram (Figure 2). Electrodiagnostic involves EMG-bilateral median neuropathy at the wrist.

**Figure 3:**
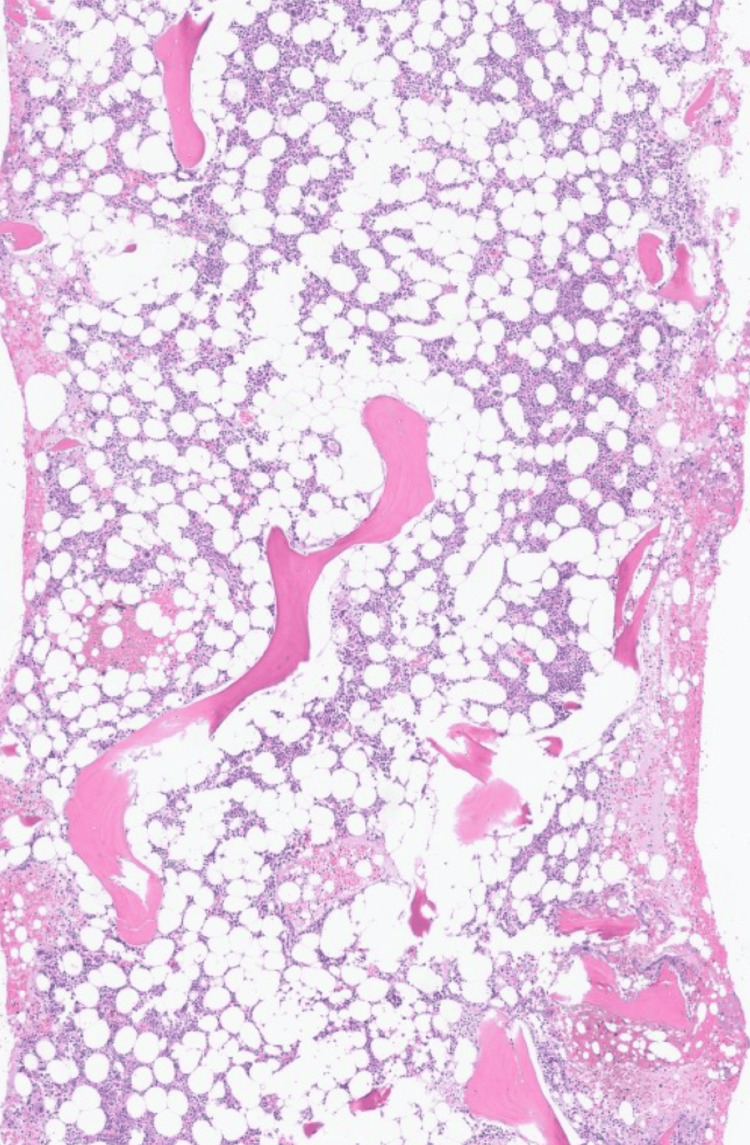
Bone marrow biopsy: morphologically normal trilineage cellularity

**Figure 4:**
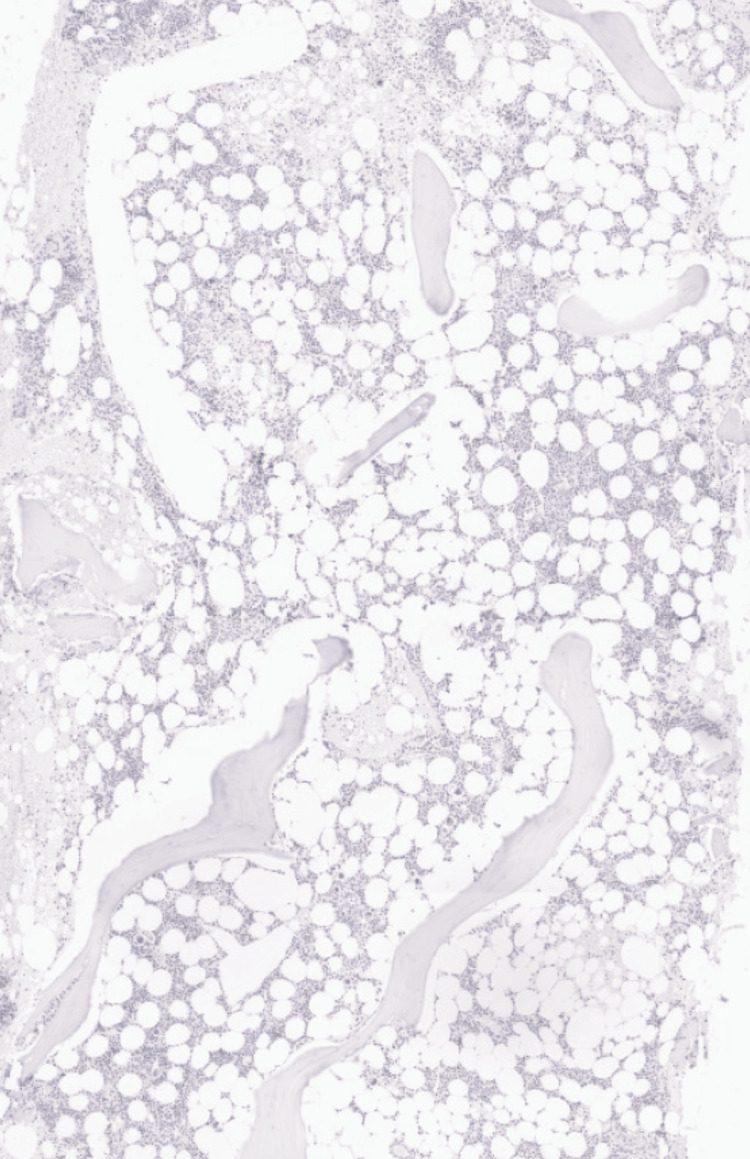
Bone marrow biopsy (Congo red stain): negative for amyloid

**Figure 5:**
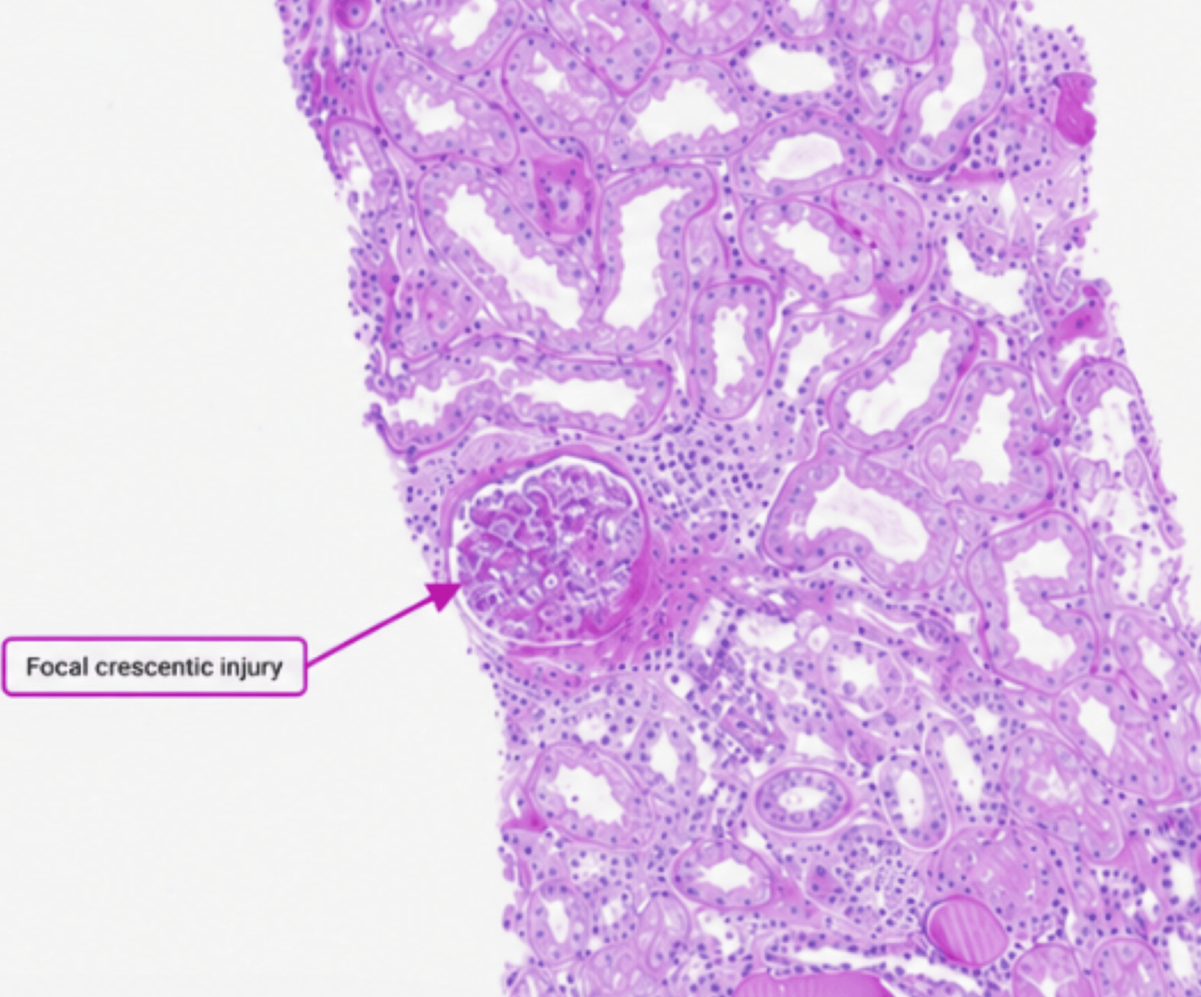
Renal biopsy: focal crescentic injury No evidence of monoclonal immunoglobulin deposition disease, proliferative glomerulonephritis with monoclonal deposits, light chain cast nephropathy, or light chain tubulopathy

**Figure 6:**
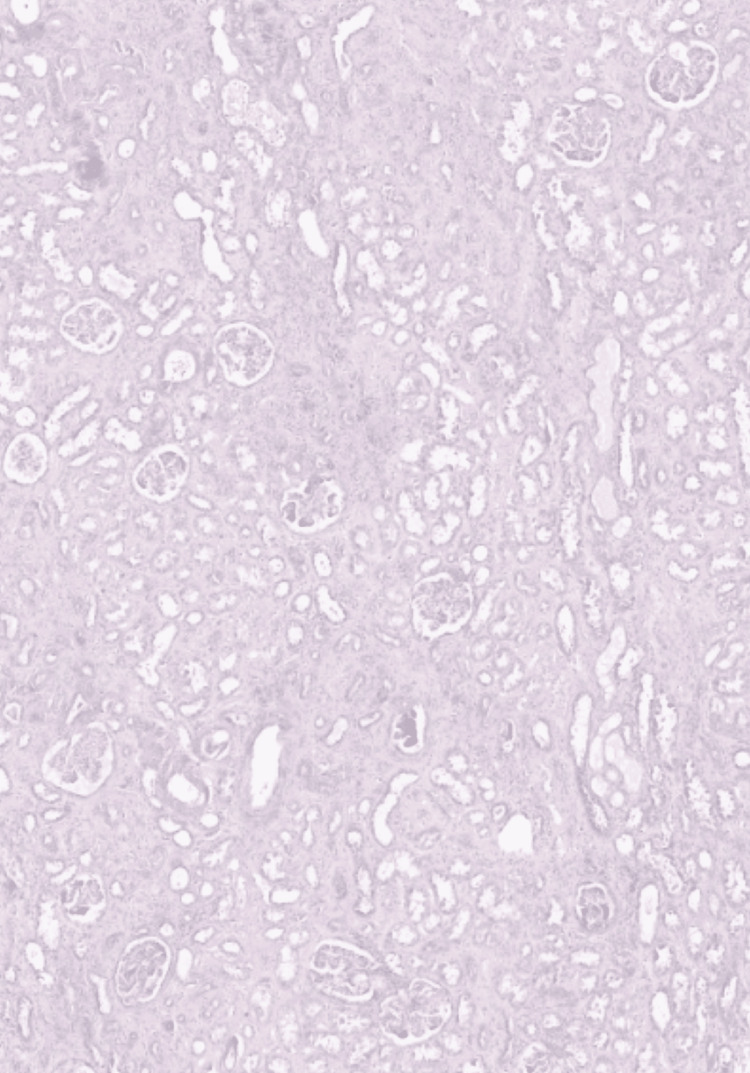
Renal biopsy (Congo red stain): negative for amyloid

**Figure 7:**
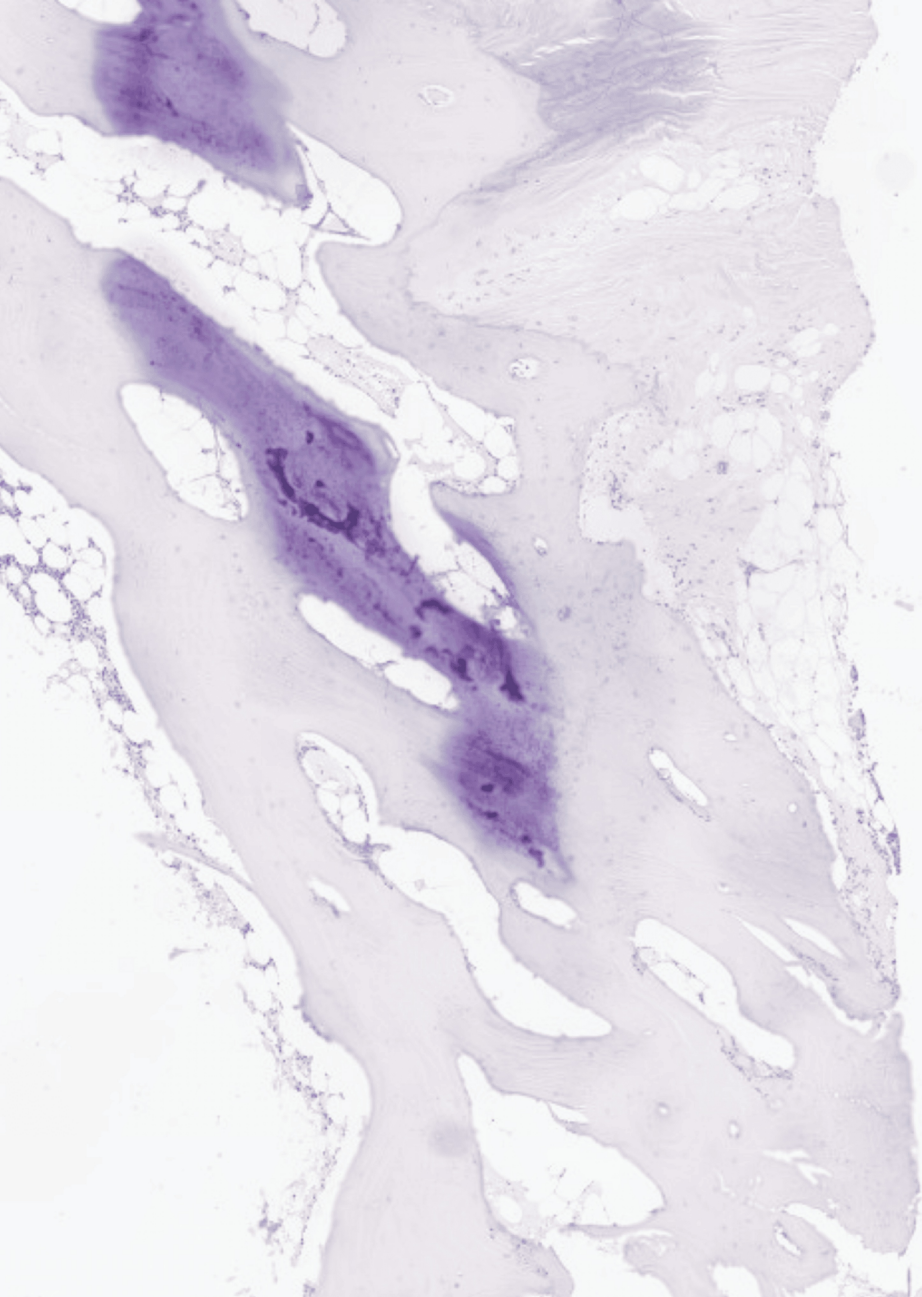
Abdominal fat pad biopsy (Congo red stain): negative for amyloid

Pathology: A bone marrow biopsy showed normocellular bone marrow with morphologically normal trilineage hematopoiesis, plasma cell proliferative disorder, 5-9% kappa light chain-restricted plasma cells (Figure 3), and negative for amyloid by Congo red stain (Figure 4). The renal biopsy was negative for amyloidosis, monoclonal immunoglobulin deposition disease, proliferative glomerulonephritis with monoclonal deposits, light chain cast nephropathy, or light chain tubulopathy amyloid (Figures 5-6). Fat pad aspiration was negative for Congo red stain (Figure 7).

Management

After ruling out other causes and based on the above workup, the patient was treated for EF.

Initial therapy: Short-course, high-dose prednisone resulted in symptomatic improvement (edema, pain, exercise tolerance). Mycophenolate was started to wean steroids to the lowest required dose or for cessation if possible. The patient refused methotrexate (MTX) treatment. EF commonly responds to glucocorticoids; MTX or mycophenolate are recommended steroid-sparing agents. Evidence suggests that initial combination therapy with systemic corticosteroids and MTX may be most beneficial. Biologic therapies have shown utility in treatment-resistant EF, often resulting in at least partial resolution of disease. IVIG and agents targeting interleukin-5 or Janus kinase pathways may be considered for refractory cases [9-11].

MGUS and MGRS: Hematology favored MGUS, with MGRS unlikely given kidney pathology, with a plan for routine surveillance of serum protein studies. The International Kidney and Monoclonal Gammopathy Research Group (IKMG) consensus supports clone-directed therapy only when renal lesions attributable to monoclonal protein are identified on kidney biopsy. Early recognition of MGRS is crucial, as suppression of monoclonal immunoglobulin secretion by chemotherapy often improves outcomes [5,12].

Outcome and follow-up

The patient was started on prednisone 20 mg and mycophenolate 1,000 mg per day after initial diagnosis. Prednisone was subsequently weaned to 5 mg per day over the next two months. The patient reported improved limb swelling, exercise tolerance, and overall well-being, as well as anemia resolved and foamy urine subjectively decreased. Her proteinuria declined from 2,976 to 354 mg per 24 hours over 12 months with normalization of albumin to 4, when rechecked in February 2026. With renal pathology negative for MGRS, Hematology planned surveillance (recheck serum protein studies at six months). Estimated GFR remained stable at 56 mL/min/1.73 m^2^.

## Discussion

EF recognition at the bedside

The groove sign and peau d'orange are classic EF clues and were both present (forearm groove sign, lower extremity peau d'orange). EF usually affects limbs symmetrically, often sparing the hands and face, and may follow exertion or travel-related physical strain [1,2].

Laboratory pointers

Peripheral eosinophilia and hypergammaglobulinemia are common but not universal; in one cohort, eosinophilia was identified in only 41.2% of patients [13].

The diagnosis of EF can be established by clinical, laboratory, and histologic findings, but universally accepted international diagnostic criteria are lacking [2]. It showed that the mean age was 44.5 years at diagnosis, revealing that the disease predominantly affects adults [14].

Imaging and histology

While a full-thickness fascial biopsy remains the major criterion in proposed diagnostic algorithms, MRI is a valuable adjunct to document fascial inflammation, select biopsy sites, and monitor treatment response. MRI reveals characteristic findings, including thickening, signal abnormalities, and contrast enhancement of the superficial and, to a lesser extent, deep muscle fasciae, with changes that correlate well with clinical findings and resolve with treatment. Our patient's reluctance to undergo biopsy prompted MRI first - an approach supported by recent multicenter studies and systematic reviews [3,15].

Hematologic associations

EF has recognized links with hematologic disorders, including monoclonal gammopathy and, rarely, overt malignancy, such as peripheral T-cell lymphoma [16]. The concept of monoclonal gammopathy of cutaneous significance has recently been proposed to describe paraproteinemia with serious cutaneous sequelae [4,5]. The pattern of paraprotein is heterogeneous; prior literature has emphasized IgG isotypes, but IgA associations are well documented across sclerosing dermatoses, notably scleroderma [5,6]. This case adds to evidence that IgA-κ MGUS can coexist with an EF phenotype. Polyclonal gammopathy is a well-described association with EF [14,20]. However, MGUS's prevalence with EF is unclear and has been described only in a few case reports.

Proteinuria and ruling out MGRS

Given the combination of MGUS and heavy proteinuria, MGRS was a critical consideration. However, a kidney biopsy showed no amyloid or monoclonal immunoglobulin-mediated lesions (e.g., monoclonal immunoglobulin deposition disease (MIDD), proliferative glomerulonephritis with monoclonal deposits (PGNMID), light-chain cast nephropathy), and renal function was mildly impaired and remained stable - arguing against MGRS and favoring MGUS with a non-MGRS kidney process or steroid-responsive inflammatory edema. By definition, MGRS is a clonal proliferative disorder producing a nephrotoxic monoclonal immunoglobulin that injures the kidney; establishing MGRS typically requires a diagnostic kidney biopsy demonstrating characteristic lesions. MGRS-related kidney lesions are classified according to the characteristics of the monoclonal immunoglobulin deposits on electron microscopy: organized, nonorganized, or absent deposits. The Renal Pathology Society and IKMG have recently established consensus-based terminology and precise definitions for monoclonal gammopathy-associated kidney lesions to harmonize diagnosis with precision therapy [5,12]. In a recent prediction tool study, 32.9% of patients with monoclonal gammopathy who underwent kidney biopsy had MGRS lesions, with amyloid light-chain (AL) amyloidosis being the most common [2].

Carpal tunnel and amyloidosis

The patient's bilateral median neuropathy raised concern for amyloidosis, as carpal tunnel syndrome is a known early red-flag sign for transthyretin amyloidosis and is associated with increased amyloidosis risk in large cohorts. Bilateral carpal tunnel syndrome in patients without medical or occupational risk factors, particularly with recurrence after carpal tunnel release surgery, is considered a warning sign of amyloidosis [8]. In wild-type transthyretin amyloidosis, noncardiac signs include bilateral carpal tunnel syndrome, lumbar spinal stenosis, and biceps tendon rupture [17]. In our case, bone marrow and kidney assessments excluded amyloid. Continued vigilance is warranted, but the absence of tissue amyloid and clinical response to steroids supported an EF-centered management plan.

Pathophysiology and triggers

An autoimmune mechanism is presumed to underlie EF, initiated by diverse triggers, and the original description by Shulman in 1974 already noted that both initial patients gave a history of strenuous exercise in the days preceding onset. Additional recognized triggers include trauma, infections, and - more recently - immune checkpoint inhibitors and vaccinations [2]. Contemporary research has elucidated a pathogenesis driven by a specific type 2 immune dysregulation involving the IL-33/ST2 axis, aberrant JAK/STAT signaling, and a distinct macrophage-to-myofibroblast transition (MMT). The process is thought to be initiated by the release of IL-33 - an "alarmin" cytokine - from damaged endothelial or epithelial cells. IL-33 binds the ST2 receptor on eosinophils and innate lymphoid cells (ILC2s), stimulating secretion of profibrotic cytokines, principally IL-5 and IL-13 [18].

Elevated levels of eosinophilic cationic protein and serum IL-5, along with increased eosinophilic migration capacity, are consistent with an augmented eosinophil count and suggest that eosinophils contribute to disease onset. Dermal fibroblasts from EF patients exhibit greater expression of type I collagen and fibronectin than healthy fibroblasts, and fibrosis is amplified by increased production of tissue inhibitor of metalloproteinases-1 (TIMP-1), an inhibitor of matrix metalloproteinase-1 (MMP-1, collagenase), limiting extracellular matrix degradation [10]. Gene expression profiling shows robust T-cell activation and cytotoxic signatures in EF despite a pauci-inflammatory histologic appearance, suggesting that small numbers of T cells may drive injury, inflammation, and fibrosis. Shared Janus kinase (JAK)/signal transducer and activator of transcription (STAT) pathway upregulation is a prominent finding across both EF and morphea. This supports your Th1/STAT1-driven IFN-γ cytotoxic T-cell arm. The Th2/STAT6-driven IL-4/IL-13 arm is corroborated by the IL-33/ST2 data above and the eosinophilic milieu. However, the precise pathogenesis of EF remains under active investigation, and the condition is still considered a disease with unknown etiology [18].

A distinct MMT has been identified as part of the fibrotic mechanism in EF. Mechanistically, TGF-β/Smad3 signaling drives this macrophage transition into collagen-producing α-SMA⁺ myofibroblasts, and M2-polarized macrophages are the primary source of TGF-β1 and platelet-derived growth factors that induce fibroblast differentiation, initiating tissue fibrosis. Elevated tissue inhibitor metalloproteinases (TIMPs) further regulate extracellular matrix deposition by inactivating matrix metalloproteinases, resulting in increased extracellular matrix accumulation in EF patients [19].

Therapeutic approach

EF typically responds to glucocorticoids; MTX and mycophenolate are favored steroid-sparing agents. Evidence suggests that initial combination therapy with systemic corticosteroids and MTX may be most beneficial. Biologic therapies have shown utility in treatment-resistant EF, often resulting in at least partial resolution of disease, and agents targeting interleukin-5 or Janus kinase pathways represent emerging options [1,2,9-11]. Multiple pieces of evidence show rituximab as an effective biologic option [11]. Physical therapy helps preserve the range of motion and prevent contractures, as decreased range of motion can significantly impact patients' quality of life [2].

Our patient's brisk steroid response and functional gains align with expected EF trajectories; most patients show favorable outcomes following steroid monotherapy or in combination with immunosuppressants [13].

In a large systematic review and meta-analysis of adult patients with eosinophilic fasciitis, 82.2% of patients exhibited a clinical response to treatment, but 24.5% of those who initially responded experienced relapse [14,20]. The largest EF cohorts studied (n=128) use multivariate correspondence analysis, where, among 109 patients, follow-up for more than one year shows that 45% patient experience relapse and 44% had sequelae as residual permanent damage. Multivariate analysis shows that eosinophilia (HR: 1.56) and fibrosis (HR: 4.02) were the strongest predictors for relapse. Relapse was managed with increased glucocorticoids and MTX, resulting in 76% clinical improvement, achieving withdrawal of 45% of patients from steroids [20]. Poor prognostic factors include marked eosinophilia and extensive fibrosis at presentation, which are associated with higher relapse rates and residual disability. Timely diagnosis and early initiation of therapy are critical, as delayed treatment and severe disease at onset are linked to worse outcomes and higher long-term sequelae [14,20].

EF in different populations

EF has an equal sex distribution, with a 1:1 male-to-female ratio. More than 80% of patients have dermatological presentations, and lower extremity involvement in more than 75%. Hyper eosinophilia is present in more than 90%, and hypergammaglobulinemia is present in more than 65% [14]. In China, a study shows that male predominance is as high as more than 70% and is frequently present with prayer signs, though eosinophilia is less in border international cohorts [13].

Teaching points: (1) The groove sign plus distal limb peau d'orange should prompt EF consideration, and high index of suspicion is necessary; (2) MRI effectively supports diagnosis and guides sampling when deep biopsy is deferred; (3) MGUS in EF is not rare-investigate for MGRS if renal involvement is suspected, but interpret pathology carefully; and (4) carpal tunnel syndrome in MGUS warrants amyloidosis screening, yet negative tissue studies can redirect focus toward EF [1-3,5,8].

## Conclusions

This case reinforces four translatable clinical principles: first, the groove sign and peau d'orange are specific enough to anchor an EF diagnosis and should prompt early rheumatologic and dermatologic evaluation; second, MRI is a clinically and evidentially justified diagnostic bridge; third, the detection of MGUS in EF obligates structured renal evaluation with kidney biopsy when proteinuria is present, interpreting pathology against rigorous MGRS criteria before initiating clone-directed therapy; and fourth, bilateral carpal tunnel syndrome in any patient with monoclonal gammopathy warrants systematic amyloidosis exclusion, though negative tissue studies should redirect rather than paralyze the diagnostic process. As universally accepted international diagnostic criteria for EF remain absent, continued accumulation of well-characterized case series and prospective cohort data are essential to refine diagnostic algorithms, prognostic stratification, and therapeutic sequencing for this rare but clinically significant fibrosing disorder.
